# Incorporating movement breaks into primary school classrooms; a mixed methods approach to explore the perceptions of pupils, staff and governors

**DOI:** 10.1186/s12889-022-14551-5

**Published:** 2022-11-24

**Authors:** Rebecca A. Chorlton, Craig A. Williams, Sarah Denford, Bert Bond

**Affiliations:** 1 Children’s Health and Exercise Research Centre, Sport and Health Sciences, College of Life and Environmental Sciences, University of Exeter, EX1 2LU Exeter, UK; 2 Bristol Medical School, Faculty of Health Sciences, University of Bristol, Bristol, UK

**Keywords:** Interrupting sitting, Classroom intervention, Physical activity promotion, Primary schools

## Abstract

**Background:**

Public health guidelines for children advocate physical activity (PA) and the restriction of continuous sedentary time. Schools offer an attractive setting for health promotion, however school-based interventions to increase PA typically fail, and primary school children may spend most of the school day sitting down. Classroom movement breaks have been identified as an attractive opportunity to address this concern and may positively influence behaviour, but little is known about the barriers to implementing movement within lessons from a multi stakeholder perspective. The purpose of this study was to explore (1) the perceptions of primary school pupils, staff members and governors regarding classroom movement breaks, and (2) their perceived barriers and facilitators to implementing PA into the classroom.

**Methods:**

Thirty-four pupils (Key Stages 1 and 2, ages 5–7 y) took part in a focus group discussion. Sixty-four staff members and twenty governors completed a questionnaire and an optional follow up semi-structured telephone interview. Qualitative data were analysed using thematic analysis.

**Results:**

Pupils, staff members and governors expressed an enthusiasm for movement breaks provided that they were short, simple, pupil-guided and performed at the discretion of the teacher. Time and concerns regarding transitioning back to work following a movement break were identified as key barriers by pupils and staff. Governors and some staff expressed that favourable evidence for movement breaks is needed to facilitate their adoption, particularly regarding the potential for improvements in cognitive functioning or classroom behaviour.

**Conclusion:**

There is a wide appeal for classroom-based activity breaks, when delivered in a manner that is not disruptive. Future research which examines the potential benefits of such activity breaks is warranted.

**Supplementary Information:**

The online version contains supplementary material available at 10.1186/s12889-022-14551-5.

## Background

Current physical activity (PA) guidelines for children and young people recommend an average of at least 60 min of moderate to vigorous activity per day across the week and call for continuous daytime sedentary activities to be minimized or interrupted [1]. Primary schools have been identified as an important setting to promote PA due to their wide exposure for policy intervention and also because this environment may account for ~ 40% of a child’s waking hours. Accordingly, schools have been encouraged to provide at least 30 min of moderate to vigorous activity per day [2, 3], however this is achieved by less than one quarter of children [4]. Furthermore, children may be more sedentary [5] and experience more prolonged sedentary periods [6] at school compared to time spent outside of school, with 50–70% of school time spent sitting [7].

Several systematic reviews have concluded that efforts to increase PA in schools typically fail [8–10]. However, such interventions usually focus on providing “extra” opportunities for PA outside of scheduled class time. Instead, targeting the prolonged sitting time inside the classroom by interrupting it with PA is an attractive approach to both increase PA and decrease uninterrupted sedentary time. Indeed classroom-based PA is specifically advocated as policy in the prevailing Comprehensive School Physical Activity Program (CSPAP) in the United States of America [11], which is widely endorsed by leading public health bodies [12, 13]. Specifically, the CSPAP provides a holistic framework to promote opportunities for PA before, after and, importantly, *during* school by engaging, supporting and empowering schools, staff and the wider community. Accordingly, the CSPAP has catalyzed much of the implementation and research regarding PA in the classroom. This includes the role classroom-based interventions may have for increasing PA [14] and minimizing prolonged sitting [15], alongside improving time on task [15–17], student behaviour [14] and academic performance [18]. Given this encouraging potential, there is a need to understand how such classroom-based movement breaks may be implemented and adopted.

The poor success rates of previous school based interventions have also been attributed to the ‘top-down’ approach utilised, whereby researchers and external parties drive intervention design with limited input from key internal school stakeholders [19]. This approach can lead to a disconnect between what is provided and what is feasible for teachers, enjoyable for pupils, and supported by senior leadership staff, head teachers (or “principals”) and school governors. In the UK school system, it is the latter who are responsible for overseeing school strategy, as opposed to head teachers and the senior leadership team who perform the day to day management, and the teachers and teaching assistants who implement such policy. Whilst many papers have reported on the perceptions of teaching staff [20–23], our understanding of the perceptions of those who oversee (governors) school policy in contrast to the teachers who implement it, and the potential tension between the two, is limited. A collaborative approach by all of these stakeholders, in addition to pupils who shape any implementation by the teacher [24], is needed to create a comprehensive understanding regarding the barriers and facilitators regarding classroom-based movement breaks. This holistic approach has been specifically called for [19, 25, 26] and is a central tenet of the CSPAP framework [11]. Previous research has mainly reported the perceptions of teachers regarding the integration of activity into the classroom, and mostly focused on the ability to integrate activity as part of the learning activity [20, 22–24, 27]. Thus, our understanding of the acceptability of movement breaks, which may be unrelated to learning, is typically limited to teaching staff. There is a dearth of information regarding the perceptions of governors, and few studies have sought to simultaneously consider the views of pupils. This study seeks to address this important gap. Specifically, the aim of the present study is to use a multifaceted approach to better understand (1) the perceptions of classroom movement breaks from a wide range of stakeholders, including pupils, school governors and across the hierarchy of school staff (teaching assistance, teachers, senior leadership team, head teachers (principles)), and (2) to identify the perceived barriers and facilitators regarding the delivery, implementation and adoption of classroom movement breaks according to these stakeholders.

## Methods

### Design

Our study was exploratory, and utilised a sequential, mixed methods design which included: (1) an online questionnaire for school staff and governors, (2) semi-structured telephone interviews with staff and governors, and (3) pupil focus groups. Data was collected between November 2019 and December 2020. Ethical approval was secured from the institutional ethics committee prior to study commencement, and all aspects of the study were in line with the Declaration of Helsinki. Teachers and governors provided written informed consent, whilst pupils and their parents/guardians provided assent and informed consent, respectively.

### Participants

Opportunistic sampling was used. The Somerset Activity and Sport Partnership assisted recruitment by initiating contact, via email, with 234 primary schools across Somerset, a county in the southwest of England. Qualitative questionnaire and follow-up interview data were collected from current primary school staff (*n* = 64) and governors (*n* = 20) from 41 different primary schools. Focus groups were conducted with 34 primary school Key Stage (KS) 1 and 2 (ages 5–7 and 7–11 y) pupils (10 boys, 24 girls) from one federated school in Somerset.

### Protocol

#### Questionnaire

Staff completed a 39-item questionnaire, and governors completed a different 41-item questionnaire using the platform Qualtrics (Qualtrics XM., Provo, Utah, USA). Staff members included Key Stage 1 (aged 5–7 years) teachers (*n* = 10), Key Stage 2 (aged 7–11 years) teachers (*n* = 12), physical education teachers (*n* = 17), teaching assistants (*n* = 7), head teachers (*n* = 12) members of senior leadership (*n* = 5) and those who described themselves as “other” (*n* = 6).

The staff questionnaire primarily focused on current attitudes, preferences and barriers surrounding the feasibility and acceptability of implementing movement breaks within lessons. The questionnaire also allowed teachers and staff to specify how often and what types of classroom PA they currently or would like to utilise. The governor questionnaire was primarily focused on governor’s main priorities when implementing change within the school environment and how likely they would be to support the implementation of classroom-based PA at a future governor meeting. Participants were given the option to leave their contact details so that they could be invited to take part in a semi-structured interview with the research team. Example questions from this questionnaire are presented as a supplementary file.

#### Semi-structured interviews

Following the completion of the questionnaire, the primary investigator contacted participants who had expressed an interest in further discussion. Individual semi-structured telephone interviews were conducted with staff and governors to clarify questionnaire responses and facilitate a deeper understanding of their perceptions regarding the value and feasibility of integrating movement breaks into the primary school classroom.

Interviews were recorded and carried out via telephone conducted by one member of the research team (RC) and lasted 15–25 min. The five interviewed staff all undertook varying roles within the primary school setting (Key Stage 1 teacher, Key Stage 2 teacher, physical education teacher, teaching assistant and head teacher), which provides insight across the breadth of school hierarchy. A semi-structured topic guide was developed for teacher interviews to ensure standardized enquiry. The topic guide is included as a supplementary file, and covered four main areas: (1) attitudes towards school-based PA, (2) current incorporation and knowledge of classroom movement initiatives, (3) barriers and facilitators of movement implementation, and (4) potential ideas for future incorporation. The order of topics and specific wording of questions altered across interviews with the aim to achieve data saturation. The researcher followed the interview guide but maximised opportunities to ask additional probing questions to allow participants to further elaborate on their answers [28, 29]. The questionnaires, topic guide and interview proceses were first piloted with three physical education student teachers and then refined before use with participants.

#### Pupil focus groups

Five focus groups were conducted, each following the topic guide presented in the supplementary file. Each focus group consisted of 6 or 7 children as recommended [30]. A mixture of boys and girls participated in each focus group to ensure heterogeneity within the groups. Focus groups were kept short (between 29 and 40 min; mean = 34 min) to ensure that children remained engaged with the discussion topics [31]. Two focus groups consisted of Key Stage 1 Pupils (aged 5–7 years) and the following 3 consisted of Key Stage 2 pupils (aged 7–11 years), as described in Supplementary Table 1. Focus groups were held at schools in vacant, quiet classrooms and were facilitated by two members of the research team. Children were positioned around the moderator in a circular position to project a non-authoritarian climate [31]. To reduce the power imbalance that can arise when an adult facilitates a children’s focus group, it was made clear the moderator was not a teacher, there were no right, or wrong answers and the children were free to express their own opinions [32]. Such strategies contribute to the credibility of the focus group data [33].

Following an ice breaker activity, an open discussion with the children was performed using a semi-structured topic guide, which featured open-ended questions exploring children’s opinions regarding; (1) the importance of PA within their school day, (2) their experiences of movement initiatives within lessons so far, including problematic elements or what went well, and (3) how attractive and successful classroom-based movement breaks might be designed. To help convey their perceptions [34], children were allowed to write or draw ‘good’ or ‘bad’ aspects about sitting down and moving in their lessons using felt tip pens and two large pieces of paper. These drawings were anonymised and used to engage the children in conversation and to clarify main concepts of the focus group, but were not analysed directly [34].

Moderators made efforts to involve quieter group members and ensure all participants were asked questions. The pupils were encouraged to express their opinions, even if these differed from peers. The moderators employed active listening, natural curiosity and allowed natural conversation between participants to flow in order to create a comfortable and informal focus group environment.

#### Analyses

Quantitative data were analyzed and presented descriptively. Qualitative data were analyzed to interpret and build on quantitative findings [35]. All interviews and focus groups were transcribed in full by the lead author (RC) and analysed using the concept of reflexive thematic analysis [36]. A semantic inductive approach was used whereby coding is directed by the content of the data and development of themes reflect the explicit content of the data. Data were uploaded and stored using the computer software QSR NVivo12 (QSR International, 2012). Following the stages of thematic analysis, codes were assigned to raw data. Initial themes were generated through examination of codes to identify clusters of similar codes, and patterns and meaning within and between codes. The team met regularly to discuss themes in relation to the existing quantitative and qualitative data set as well as existing theory. Themes were then checked against the data set to ensure the themes provided a persuasive account of the data. At this stage, the research team looked for potential patterns in occurrence of themes within and between the three participant groups and explanations sought for any instances in which occurrence of themes differed between the groups. In the final two phases, themes were named and defined. Multiple strategies were used to enhance transparency and rigor. This included prolonged engagement with the data, keeping a clear audit trail of the process in which ideas about potential codes, themes, patterns and relationships were noted, triangulation of data against quantitative data and theory, and active seeking of disconfirming cases.

## Results

### Importance of PA

Most staff members (53/58) and all head teachers within this study valued PA for children as ‘highly’ or ‘extremely highly’ in the questionnaire, although they provided contrasting reasons. Key supporting quotes from the follow up interviews are provided in Supplementary Table 2. The most common supporting reason for valuing PA in the questionnaire was to promote the day to day physical and mental wellbeing of pupils, as highlighted by a member of the senior leadership team *“physical development is a prime area and is age critical in the development of young children”*. Many staff also commented on the benefits of PA inside of school to stimulate the pupils to assist and reinforce learning, improve behaviour and to aid refocusing. One teacher felt that *“children sitting still for long periods of time has a negative effect on learning and being active for a minimum of 60 minutes is something we value”*.

Five staff members, including Key Stage 1 teachers (*n* = 2), a Key Stage 2 teacher, PE teacher and a teaching assistant, valued PA as ‘low’ or ‘moderate’. A major theme for the lower value placed on PA was ‘time’. A Key Stage 2 teacher who valued PA as ‘low’ stated that *“there are lots of other things that come above PA”*. These individuals also expressed concerns that PA could reduce quality of learning *“It’s good for the children to be up and moving, completing practical tasks. However, it is also important they have a full understanding of what they’re being taught which might consist of simply listening”* whilst a teaching assistant perceived PA as ‘moderate’ due to *“lack of space and safety”* and many classes include children with behavioral needs who *“can become either overexcited or distressed with such activity”*.

All Governors within this study (*n* = 20) valued PA for children both in and outside of school as high or extremely high. The main reasons given for this included perceived benefits to mental and physical health and behaviour. PA was also considered to be a positive determinant of future health, important for personal and social development, critical to aid focus and learning, and offer relief from sitting at a desk. Governors were generally in support of utilising classroom movement opportunities with one Governor stating that *“any corrective intervention that will help this nation reduce a tendency toward obesity and long term physical and mental health conditions needs to be integral to education at the earliest opportunity”.* Academic results and Ofsted (Office for Standards in Education) inspections featured most commonly as the Governors’ lowest priorities.

The pupils’ perceptions are provided in Supplementary Tables 1, in pseudonymised form (“P” = participant). Most pupils felt that being physically active in the classroom was important as *“it could improve our brains…if we understand the work better”* (P5, Group 4, Key Stage 2) and *“sitting down all lesson is really not good for your health*” (P4, Group 4, Key Stage 2*)*. However, some pupils did express that they would prefer to be sat down for the entirety of the lesson as breaks could interrupt learning. For example, one pupil said *“I get distracted when I have to get up and move”* (P3, Group 2, Key Stage 1) and another preferred sitting down as *“it’s calmer”* (P7, Group 2, Key Stage 1). In contrast, most pupils mentioned feeling stiff, sad, bored and tired when sat down for long periods. A pupil stated that they *“learn less sat down”* (P1, Group 5, Key Stage 2). Some pupils felt extremely frustrated when describing how they felt when sat down all lesson with one pupil stating they feel *“limited*” (P5, Group 2, Key Stage 1), whilst another drew a picture of a cage and stated they *“feel locked away when sat down all the time”* (P1, Group 2, Key Stage 1).

### Current PA practices

Most staff members reported incorporating some form of activity in their lessons ‘once a week’ (*n* = 15) or ‘1–2 times a week’ (*n* = 14). Forty-five staff members stated that they would like to incorporate more PA into their classroom or school. Despite the value placed on PA by most staff members, eight stated that they ‘never’ incorporate PA, three of these were Key Stage 2 teachers.

The majority of staff were aware of, and had used, PA initiatives in their own lessons (*n* = 50) compared to those who had not (*n* = 8). The most popular initiatives used included Wake and Shake, Go Noodle, Fitter Futures, Joe Wicks®, BBC Super Movers, Active Math’s, Take 10 and the Golden Mile®. Many staff members who incorporate movement breaks utilise ‘videos with music/activities they can follow”. Of those staff members who used PA initiatives, most individuals found them ‘extremely useful’ (*n* = 23) or ‘moderately useful’ (*n* = 19).

In line with the staff responses, most pupils identified “videos in the classroom” as the main form of movement they have tried in their lessons, in particular *“Go Noodle”, “Be active”* and *“Just Dance”.* Other pupils have tried *“star jumps”, “jogging in one space”*, *“yoga/stretches”, “copying the teacher’s movements”* and *“wake and shake”*. Pupils cautioned that movement interventions *“need variety” and should be “something where everyone is involved and moving the whole time”* (P4, Group 5, Key Stage 2). For example, one pupil mentioned that *“it can get quite repetitive on an easy video after a while”* (P7, Group 5, Key Stage 2) with another stressing that *“it is hard to find videos both girls and boys like…some people don’t like dancing and people feel self-conscious”* (P5, Group 4, Key Stage 2). Types and format of PA appeared important, with one pupil highlighting that *“sometimes after, people go a bit crazy and start jumping around and not listening, people settle down slowly if the video is too energetic”* (P2, Group 3, Key Stage 2), on the other hand, another pupil described the opposite effect as “yoga made me sleepy” (P7, Group 4, Key Stage 2).

Most Governors were not aware of PA initiatives and did not know of any currently being used in their school, which likely reflects that the Governor role is rarely involved in the day-to-day operational side of the school.

### Experience of incorporating PA into the classroom

Pupil enjoyment of classroom movement breaks was reported by all staff members in this study. A Key Stage 1 teacher noted that *“children are generally very excited when doing PA, they are motivated and engaged”* they particularly enjoy a *“a break from the norm”* the *“spontaneity”* and *“child led elements”“They enjoy the chance to dance, stretch and move away from sitting in chairs”.*

Most staff within this study agreed that classroom movement breaks have a moderately positive (*n* = 24) or extremely positive (*n* = 20) impact on pupils because of its perceived benefits for pupil’s attention and readiness to learn. Relevant comments included: *“after the PA their concentration improves and they’re in a better place ready to learn”* (Key Stage 1 teacher), *“the child I look after can listen and concentrate better for the next 10/20 minutes”* (teaching assistant), *“pupils are generally more productive which leads to an improved attitude to learning and less poor behaviour”*. A head teacher highlighted that *“As a general rule, if implemented and managed properly, this kind of intervention definitely leads to a sustained period of concentration and good behaviour”*.

In contrast, two staff members reported experiencing an overall negative experience of incorporating PA into the classroom. A Key Stage 2 teacher cautioned that movement breaks *“can wind them up too much if it’s been energetic…it can make them too hyper and can be difficult to bring them back down for learning”*. A teaching assistant who stated the incorporation of PA had an ‘extremely negative’ effect on their pupils further supported this as *“it takes too much time for the children to settle back down to focus. Once settled, their concentration and attention is much the same as before. SEN[special educational needs]and behavioral needs children need extra time after to calm, some need up to 30 mins before they can return to task”*. A consensus among teachers was that following movement breaks *“it can take a while to settle back down to a concentrated work level”* however, once settled it generally shows positive affects in terms of pupil’s concentration, focus and behaviour.

Most pupils felt that they learnt better after a movement break as they could *“concentrate on work”* (P3, Group 3, Key Stage 2), *“think a bit clearer”* (P4, Group 3, Key Stage 2) and felt *“more awake”* (P3, Group 2, Key Stage 1). One pupil drew a picture of a brain with a smiley face in it. One pupil said they felt *“powerful”* (P2, Group 3, Key Stage 2) when they danced, with another reporting feeling more *“motivated”* and *“energetic”* (P2, Group 3, Key Stage 2) afterwards. Most pupils also agreed that their learning was worsened when sat down for the whole lesson, *“I lose interest in what the teacher is saying”* (P2, Group 5, Key Stage 2), and *“mind goes blank”* (P5, Group 5, Key Stage 2). All pupils agreed that theirs, or their classmates, behaviour deteriorated when they were sat down for too long, relevant comments included *“people start flicking pens”*, *“get distracted”* (P3, Group 4, Key Stage 2), *“drift off into your own world instead of listening”* (P2, Group 4, Key Stage 2), *“I wiggle a lot”* (P4, Group 4, Key Stage 2). Alternatively, some negative aspects of movement breaks were highlighted as *“people settle down slowly if the video is too energetic*” (P3, Group 5, Key Stage 2), and *“sometimes after Just Dance people are crazy and start dancing around and not listening”* (P2, Group 4, Key Stage 2). One pupil stressed that movement breaks are an investment for teachers as “*it would take a bit of time afterwards but it helps you concentrate and think a bit clearer so it saves time afterwards”* (P4, Group 3, Key Stage 2).

### Barriers to implementation

Staff members (*n* = 22) most commonly found it ‘moderately easy’ to integrate movements into the classroom with thirteen staff members finding it ‘neither easy nor difficult’ and eleven finding it ‘moderately difficult’. However only four staff members found it ‘extremely easy’ to implement movement into the classroom.

The three most prevalent barriers that staff members reported were time constraints, curriculum demands and perceptions regarding the ease of transitioning back to work following any movement break. Of these reasons, the foremost barrier was time, and this was directly related to curriculum pressures. A Key Stage 2 teacher, who found the incorporation of PA as ‘moderately difficult’, stated that the *“Ever increasing demands of the curriculum meaning time is precious. Having taught for 7 years, I’ve seen a dramatic shift towards filling every spare minute with some additional learning”*. One Key Stage 1 teacher shared their frustration *“time, it’s always time, there’s never enough time”*. This was supported by a head teacher as *“in an already crowded curriculum there are so many expectations on what a school should be now”.* Conversely, one head teacher disagreed *“the most effective strategies really don’t take much time. This is not a massive barrier”*.

More staff described themselves as moderately confident (*n* = 25) than moderately unconfident (*n* = 7) about implementing movement breaks in the classroom. They felt their school could do more to support this, by allocating more time for movement, utilising workshops to promote activity with core subjects, and offer awards. A head teacher reflected that staff might perceive incorporating movement breaks as an unnecessary risk, *“because results at primary school matter, people will naturally take the safest option and sometimes that’s the head teachers fault”.*

Most governors shared teacher concerns and felt unsure whether movement breaks would disrupt the class or enhance their learning, but would be supportive of movement breaks if favourable evidence were presented *“you’ve got a finite amount of teaching time so you have to justify that…. if you can show that actually standing up and moving periodically improves concentration and therefore helps your learning I can get behind that argument”*. Data regarding the effect on pupil behaviour, engagement and learning was highly valued, but Governors differed in their views regarding the nature of this supportive evidence. One Governor felt that *“a report from the class teacher should be enough, detailing how the intervention impacted on the class and how it improved engagement and concentration in class”*. In contrast, one Governor stated that *“we’d probably want to see that it has worked somewhere else first as it would be quite disruptive to implement something that didn’t work out”*.

### Recommendations for the sustainable adoption of movement breaks

There was a consensus between all stakeholders that future delivery of classroom movement breaks should occur using a variety of online resources. For example, a head teacher stated that their school classrooms *“had utilised Just Dance via YouTube which seemed to have longevity”.* A Key Stage 1 teacher agreed *“there’s so many online things that you can whip out, it seems more interesting for the pupils to watch a video”*, and added that *“having these programmes is useful, they’re free, easily accessible as everybody has interactive whiteboards in their classrooms”.* Readily available resources were identified as being important for more reluctant staff to engage with movement breaks. A Key Stage 2 teacher commented *“before I discovered Go Noodle there were some teachers not sure of what to do and having the confidence to do it”*, on the other hand, she did stress that *“it is only useful if you have a good interactive whiteboard and screen that works as I know some classrooms don’t have that”.*

All staff members agreed that future interventions should include the pupils in decision making process. For example, a Key Stage 2 teacher stated that *“it is good to give them a choice and ownership so they’re more interested but it’s still within the teacher’s control”*. When asked what they would like to see in future classroom movement breaks the pupils were positive about using videos and music chosen by them but valued the control of the teacher. Relevant comments included *“music is best”* (P3, Group 2, Key Stage 1), *“we can have fun copying videos”* (P5, Group 2, Key Stage 1), *“I would rather the teacher tell me what to do because it works better”* (P7, Group 3, Key Stage 2) and *“we could take it in turns choosing the videos”* (P6, Group 4, Key Stage 2).

When asked which periods of the day would be best to incorporate PA, the majority view was that there should be no set time for PA as *“it depends on the pupils, the activity and the teacher to be most effective”* and should be used *“with the teacher’s judgment of how they integrate based on how the class is performing, behaviour or well-being”.* A PE teacher advised *“there’s not one size fits all…if you want maximum impact you have to put it into the hands of the class teacher to choose their timing”*. A head teacher agreed with this view and stated that *“letting the teacher pick the time point of the activity within their lesson would be perfectly realistic. I think as an experienced teacher you can read your class physicality…and see clearly when they may need a movement break”*. There was no consensus regarding when pupils thought movement breaks would be best, for example . (P3, Group 1, Key Stage 1), “*I feel a bit tired after lunch so copying videos would make me feel better*” (P7, Group 2, Key Stage 1). Most pupils agreed that it depends on the day, topic and how long they have been writing/listening for, and agreed that any activity should be performed at the teacher’s discretion when he/she notices them becoming tired.

Most staff who have utilised classroom movement breaks felt that *“in terms of breaking up lessons, 5 minutes is a realistic amount”.* A Key Stage 1 teacher stressed that *“5 minutes is just the perfect amount of time as it doesn’t eat too much into your lesson time and you don’t have to leave the classroom to do it…something you can do quickly in the classroom”.* This aligned with the feedback from pupils, who identified that 2–7 min of movement is appropriate, with 5 min as the optimum length per lesson. One pupil said *“my legs get tired when I stand up for too long…if its minutes I will do it”* (P6, Group1, Key Stage 1). One pupil disagreed and felt that slightly more than 5 min may be beneficial as *“sometimes we do 5 minutes or 2 minutes and it just doesn’t feel very long so I think maybe like 7 minutes would be better”* (P7, Group 3, Key Stage 2).

The majority of Governors stated that they, and their fellow governors, would be moderately to extremely likely to support the implementation of such movement break ideas highlighted by staff and pupils in this study. Despite this two Governors disagreed by stating they would be moderately unlikely to support a classroom-based movement intervention as they felt that, although PA had a place in the school day, they were sceptical about its benefits in the classroom / curriculum.

## Discussion

The purpose of this study was to provide a greater understanding of the perceptions regarding classroom based movement breaks in primary education from a range of key stakeholders, and their perceived barriers and facilitators, which will be valuable in informing future implementation. We report here that the majority of staff (across the school hierarchy) and pupils had positive perceptions of classroom based movement breaks. Our findings also indicate that there is an appetite for their wider adoption, and that this may be supported by school governors. Studies addressing the acceptability of future classroom activity breaks, and their impact on valued outcomes such as behaviour and academic performance, are now warranted.

Our data indicate that most primary school staff, pupils and governors are supportive of classroom based activity breaks, whilst many staff highlighted a desire to include more movement breaks. In line with previous research [37], this appears to be driven mostly by a perceived link between interrupting continuous sitting time and improvements in sustained concentration or classroom behaviour, rather than concerns regarding health promotion. Specifically, staff (and pupils) identified that the ability to concentrate can be improved following an activity break, when appropriately delivered. Such data support a growing body of evidence which suggests that interrupting a class with an unrelated movement break might benefit the ability to remain on task [14, 18, 38]. This appears to highly valued by the staff in this study, and may be a key driver for the wider adoption of movement breaks [20, 39]. Further study is needed to explore the potential of movement breaks to favourably influence classroom behavior, particularly to convince school leadership teams and governors that such time taken “away” from a lesson is worthwhile [40].

Our findings indicate that the increased availability of technology in classrooms and online resources available have made the delivery of movement breaks more feasible and palatable to teachers. Such technology likely diminishes the role of teacher confidence as a barrier to implementing some activity, which has been raised regarding active learning [21, 41]. Additionally, a movement break can be, by design, a break from learning, which offsets the need to plan how activity can be effectively integrated as part of a lesson. Consequently, interrupting, rather than complimenting learning with some activity, might be the preference of some teachers given that little expertise appears to be needed.

A key staff barrier to implementing, or increasing the provision of, movement breaks was a sense of limited time and competing demands of the curriculum, which is consistent with existing studies across different countries [42]. However, arguments were made that brief (≤ 5 min) bouts of activity, performed at the teacher’s discretion and including some element of student choice would be feasible for staff and enjoyable for pupils. Some staff identified a concern that a school might not be supportive of such activity. However, there was support across our sample of head teachers and members of school senior leadership teams. Governors also expressed an enthusiasm for movement breaks, but would be more supportive in the face of favourable evidence, particularly regarding the longevity of classroom activity breaks, and whether outcomes such as classroom behavior or cognition can either be maintained or improved. This evidence appears to be the necessary precursor for wider adoption from the perspective of Governors. However, we report here that both staff across the school hierarchy (including those that do not teach physical education) and Governors appear to be supportive of implementing activity breaks, which is encouraging given the appetite demonstrated by the pupils. Interventional studies are now needed to determine whether benefits to behavior and/or cognition are possible.

A key strength of our study is that staff and pupils contributed recommendations regarding how an activity break could be successfully implemented. Our data indicate that staff should have autonomy regarding when (and if) a movement break should be delivered, which is in line with recently published work [27]. Importantly, this view was also echoed by the Governors and head teachers. Supporting staff to take ownership of this decision making is likely to be crucial to the longevity of any initiative to include activity breaks. Thereafter, our data from pupils and teachers indicate that a break of up to 5 min in length appears to be optimum, with students being provided some level of choice from a bank of readily available resources. We believe that the provision of resources (such as Go Noodle), or access to online music or videos, may also help the delivery of such activity breaks. We hope that future interventional designs will consider these recommendations, in the hope that their co-design by pupils and teachers will improve the acceptability of classroom based activity to both parties, and more easily implemented into the existing school day. These interventions should be accompanied by implementation outcomes and process evaluations in order to understand their longevity.

Whilst our focus on activity breaks compliments the growing area of “active learning”, our findings should be considered against a number of limitations. Firstly, we were not able to include the perceptions of parents and carers in this work as a “fourth stakeholder”. Such approval and support may influence a school’s decision to routinely incorporate movement breaks. Indeed, the role of family in the adoption of PA is specifically identified in the CSPAP framework [11]. To this end, discussions with local and national educational authorities will also become more pertinent in the future. Secondly, our data were exclusively collected in the South West of England, which has the highest percentage of physically active adults and children in England [43]. It is possible that the specific value a member of staff or governor places on PA will influence their desire to incorporate movement breaks [20, 44]. With this in mind, it is important to acknowledge that response rates for staff member questionnaire data were low (64 staff members from 41 different primary schools), which may impact on the generalisability of findings to all primary school staff members, and potentially bias towards individuals who more highly value PA. However, there was evidence of having achieved data saturation in open-ended responses in that no new knowledge and/or themes emerged as the number of responses analysed increased. One consideration is that the data may have been subject to sample or interview bias. For example, almost a quarter of the staff who responded to the questionnaire identified as a “physical education teacher”. However, we did not find that the perceptions of staff regarding classroom PA were constrained by job role, and it their insight is of value when considering what sorts of activity might be feasible within a conventional classroom setting. Additionally, interviewees may have been providing information they thought the researchers wanted to hear, however due to the impartial interview techniques used, the researchers do not believe this impacted the study findings. A final limitation is the exploratory nature of this work. Our goal was to simultaneously understand the initial perceptions of classroom-based PA across these key stakeholders. To this end, we now call for implementation outcomes to be considered alongside any formal design of classroom-based PA in order to capture the acceptability of future intervention designs according to these groups, preferably with the additional consideration of parents. Such studies should also be accompanied by appropriate process evaluation to provide insight regarding their feasibility and potential longevity.

## Conclusion

Our findings demonstrate that classroom-based PA breaks may be attractive from a multi-stakeholder perspective. Specifically, there is wide enthusiasm for PA breaks from those who would steer (Governors), manage (head teachers and senior leadership team) deliver (teachers and teaching assistants) and receive (pupils) such a change in school policy. We also provide suggestions for how such breaks might be successfully implemented in the future. Further work is required to scrutinize the sustainability of activity breaks, which will likely be driven by favourable evidence regarding classroom behavior.

## Supplementary information

**Additional file 1.** Questionnaire – Example questions.

**Additional file 2: Supplementary Table 1.** Supporting comments from the pupil focus groups.

**Additional file 3: Supplementary Table 2.** Key supporting quotes from staff during follow up interviews. “Q” identifies that the quote came from questionnaire data. Each quote is provided from a different individual.

## Data Availability

All supporting data are provided in supplementary files, however raw data (such as full interview transcripts) are not able to be shared as this was not specifically listed in the consent form.

## Abbreviations

PA: Physical activity
