# Supplementary material for: Incorporating movement breaks into primary school classrooms; a mixed methods approach to explore the perceptions of pupils, staff and governors
Source: BMC Public Health. 2022 Nov 24;22:2172. doi: 10.1186/s12889-022-14551-5 (PMC9701018; doi:10.1186/s12889-022-14551-5)
Supplement: Supplementary file 1 — Additional file 1. Questionnaire – Example questions. [file 12889_2022_14551_MOESM1_ESM.docx]

| **Focus group** | **Sitting down** | **Movement breaks** |
| --- | --- | --- |
| **1**  **Key Stage 1**  **(Age 5-6)** | “Writing makes me tired” (P6)  “Bored and not active” “running and playing with friends” (P4)  “I am tired in the morning and want to stand up” (P3)  “stiff” (P5)  “I want to get up for a little bit” (P6) | “tired” “running” (P1)  “jog” “jump” “happy” (P2)  “I like doing exercise because it makes you fit and makes me happy. I felt healthier after” (P4)  “10 seconds” “I felt tired” (P3)  “happy” “wakes me up” ‘outside” “enjoyed it” “tired if long amount of time” (P5) |
| **2**  **Key Stage 1**  **(Age 6-7)** | “legs start to hurt” (P6)  “trapped” “angry” “bored” “tired” *drew a picture of a cage* (P1)  “not relaxed” “tired” (P4)  “get a stiff bottom” “better and calm” (P3)  “stiff and bored” “pained” “limited” “learn worse” “stressed” (P5) | “distracted if moving” “wake and shake” “I feel free” (P6)  “wake and shake” “music is good” “yoga” *brain and happy picture* “stretches” “relaxed” “wake and shake every day or more” “happy” (P4)  “lots of sport energizes” “happy “hot” “at least 2 minutes of activity” “having fun” “music” (P3)  “chilled” “5 minutes” “excited for the few minutes” “stretches ready for the day” (P5)  “enjoy myself” “doing active things that make us all happy” “doing fun things like music” (P2)  “copying videos” “I feel free” “being awake” (P1) |
| **3**  **Key Stage 2**  **(Age 7-9)** | “tired” “no fun” “bored” “noisy” (P4)  “Chairs are boring” “get hungry” “pins and needles” “get sad” “rather be at home” (P3)  “want to do something else” “boring’ (P1)  “I start chatting” (P2)  “would like to listen to music” (P5)  “hard to concentrate” “restless” “want to start chatting” (P6) | “stretch your fingers” “wake and shake” “5 minutes” “better” “lively” “just dance” “10-15mins active” “wake and shake” “5 mins” (P3)  “helps concentrate” “jogging on the spot” “do some stretches for 5 minutes” “yoga is boring” “Simon says” (P5)  “after lunch” “yoga for 5 mins” “energetic dance” “lively” “just dance” “enjoy it a lot” “young leaders lead wake and shake” “7-13mins” (P4)  “Wake and shake” “bean bags” “go noodle” “dance powerful” (P1) |
| **4**  **Key Stage 2**  **(Age 9-10)** | “bad health” “eyes sore” “upset mind” “zone-out” “headache” “bored” (P5)  “no fun” “slouch” “dull” “want to get up and talk” (P2)  “unhealthy” “go into own world instead of listening” “you feel like you’re trapped in a box” (P3)  “not good for health” “boring being silent” “neck aches” “zoning out” (P4)  “sounds like the teacher speaks nonsense” “lost” “day dreaming” “hard to concentrate” (P1) | just dance” “more learning” “go noodle” “wake and shake” “when they saw us getting a bit tired” “helps motivate us” “helps you learn more” “music” “about 3 mins” “two different activities” (P2)  “boys get embarrassed” “go noodle” “just dance” “helps more” “wake and shake” “2 mins” “it makes lessons more fun” “yoga” “makes you smile” “athletic” “happier” (P1)  “it gets into my head more” “5 minute is the right amount of time” “just dance” “go noodle” “feel happier” (P3)  “happier” “go noodle would be good” “just dance” “going outside more” “middle of the lesson breaks” “more exciting” “helps you remember” “about 2 mins every lesson would be good” “wake and shake” “energetic” “copy the screen” (P4)  “just dance” “happy!!” “cramped” “5 minutes” “1-2 mins is ok” “more wake and shake” “let others choose something to do” “go noodle” (P5) “motivated” “yoga makes you tired” “helps you learn” “help you to improve your memory of what you are doing” “movement is good” (P6) |
| **5**  **Key Stage 2**  **(Age 10-11)** | “get bored” “legs ache” “I start to talk” “forget what the teacher is saying” (P1)  “uncomfortable” “lose focus” “can’t think” (P3)  “BOREDOM!” “get tired” “get distracted by random thoughts” (P4)  “I begin to fidget” “I feel like sleeping” (P2)  “do more movement” “get bored” (P5)  “I begin to fiddle with my pencil and stare into space” (P6)  “it gets boring” “my mind goes blank” (P7) | “every half and hour activity break” “15 min break” “variety” “copy the teacher” (P1)  “go noodle” “more wake and shake” “games” (P3)  “I feel excited” “it gives me more motivation to write” “I work harder” “go noodle” (P5)  “13 mins” “after we feel much more energetic and ready to learn” (P4)  “10 mins” “in the afternoon” (P7) |

**Supplementary Table 1:** Supporting comments from the pupil focus groups
