# Supplementary material for: Incorporating movement breaks into primary school classrooms; a mixed methods approach to explore the perceptions of pupils, staff and governors
Source: BMC Public Health. 2022 Nov 24;22:2172. doi: 10.1186/s12889-022-14551-5 (PMC9701018; doi:10.1186/s12889-022-14551-5)
Supplement: Supplementary file 2 — Additional file 2: Supplementary Table 1. Supporting comments from the pupil focus groups. [file 12889_2022_14551_MOESM2_ESM.docx]

| **Theme** | **Subthemes** | **Supporting quotes** |
| --- | --- | --- |
| **PA importance** | Stimulate pupils and reinforce learning  Physical and mental health  Age critical nature  School day – Sedentary | *“I believe physical activity in the classroom creates the perfect conditions for individual learning”* (PE teacher)  *“physical development is a prime area and is age critical”* (Senior leadership Q)  *“physical activity is key to the health of children in schools. As health is considered to be deteriorating in young children, getting them involved in physical activity from an early age is key to future health”* (KS2 teacher Q) |
| **Use of initiatives** | Example initiatives  Resources used | *“YouTube”, “videos with music/activities they can follow”* (KS2 teacher Q) |
| **Current PA practices** | Frequency of use  Timing of use  Length of movement break | *“it depends on the pupils, the activity and the teacher to be most effective”* (Headteacher Q)  *“if you want maximum impact you have to put it into the hands of the class teacher to choose their timing”* (Headteacher)  *“in terms of breaking up lessons, 5 minutes is a realistic amount”* (KS1 teacher) |
| **Barriers to incorporation** | Space/safety  Time constraints  Curriculum demands  Sustainability of initiatives  Fear of disruption/transitioning back to class work  Teacher willingness | *“Ever increasing demands of the curriculum meaning time is precious”* (Headteacher Q)  *“space in schools as quite a big issue as well as time and expectations of lessons and fitting everything into the day”* (KS1 teacher)  *“some teachers are a little bit nervous about it as then you don’t have as tight control over your class”* (KS2 teacher)  *“some staff are stuck in a traditional routine”* (Headteacher) |
| **Experience of incorporation** | Over excitement/settling back down  Concentration  Energised  Behaviour | *“after the PA their concentration improves and they’re in a better place* *ready to learn, “it energizes them”* (KS1 teacher)  *“it can wind them up too much if it’s been energetic” “can take a while to settle back down to a concentrated work level”* (KS2 teacher) |
| **Intervention ideas** | Resources  Pupil led | *“having these programmes is useful, they’re free, easily accessible”* (KS1 teacher)  *“a little bank of resources that you can pull out and use each day would be useful”* (KS2 teacher)  *“pupil led…semi organic….short bursts…still within the teachers control”* (PE teacher) |

**Supplementary Table 2:** Key supporting quotes from staff during follow up interviews. “Q” identifies that the quote came from questionnaire data. Each quote is provided from a different individual.
