# Supplementary material for: Incorporating movement breaks into primary school classrooms; a mixed methods approach to explore the perceptions of pupils, staff and governors
Source: BMC Public Health. 2022 Nov 24;22:2172. doi: 10.1186/s12889-022-14551-5 (PMC9701018; doi:10.1186/s12889-022-14551-5)
Supplement: Supplementary file 3 — Additional file 3: Supplementary Table 2. Key supporting quotes from staff during follow up interviews. “Q” identifies that the quote came from questionnaire data. Each quote is provided from a different individual. [file 12889_2022_14551_MOESM3_ESM.docx]

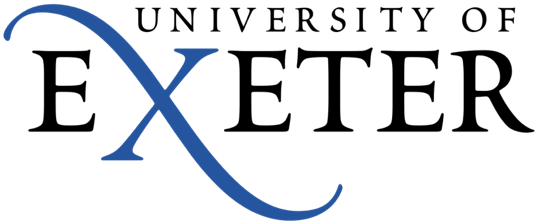

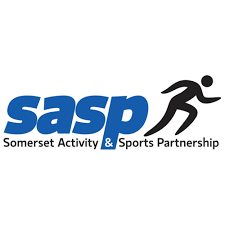


**Questionnaire – Example questions**

Q19 – How highly do you value the role of Physical Activity in the classroom?

*Extremely low*

*Low*

*Moderate*

*High*

*Extremely high*

Q20 – Please explain your answer

Q28 – Could you briefly explain why you have / have not used any physical activity initiatives in your lessons?

Q27 – How often do you currently incorporate movement into your lessons?

*Never*

*1-2 times a week*

*3-4 times a week*

*Once a day*

*2+ times a day*

Q30 – At what periods in the lesson or school day do you feel it is best to implement physical activity? (tick all that apply)

*End/ between lessons*

*Start of lesson*

*Middle of lesson*

*Morning lessons*

*Afternoon lessons*

Q34 – What do you think are the main barriers to increasing physical activity in the classroom / school setting? What is making it difficult for you to include movement in the classroom? (you may wish to comment on resources, time, other staff, parents, training, child behaviour, feasibility or curriculum time).

Q37 – How confident are you using movement activities in the classroom?

*Extremely unconfident*

*Moderately unconfident*

*Neither confident nor unconfident*

*Moderately confident*

*Extremely confident*

Q38 – What are your experiences of how movement activities would affect your students and the classroom atmosphere?

*Extremely negative*

*Moderately negative*

*Neither positive nor negative*

*Moderately positive*

*Extremely positive*

Q39 – Please provide more detail behind your rating above in terms of:

*pupil concentration*

*time spent on task*

*and pupil behaviour* following the movement break.

**Semi structured topic guide example questions – staff interviews**

**Q1 - How important do you think breaking up prolonged sitting with physical activity is for children?**

1. *Why?*
2. *Do you feel that academic achievement is too much of a priority in your school at the expense of physical activity?*
3. *What is do you think your head teacher’s opinion is on this?*

**Q2 - Are you aware of any physical activity initiatives to help teachers build movement into their lessons?**

1. *Please name a few and tell me more about them.*

**Q4 - Did you find any of the initiatives you have used useful?**

1. *If so what elements were most useful and why?*
2. *If no, why were the initiatives not so useful to you?*

**Q5 - If you currently incorporate movement into your lessons how often do you do this?**

**Q6 - At what time points across the school day do you feel is best to implement the physical activity?**

1. *Why?*
2. *What time points do you feel it would be best to avoid and why?*

**Q8 - If you implement physical activity into the classroom how long does each break last for approximately, and why?**

**Q9- How easy or difficult is it to integrate movement into the classroom?**

1. *What are the main barriers to increasing physical activity in the classroom or school setting? (You may wish to comment on resources, time, other staff, parents, training, child behaviour, feasibility or whether this might impair curriculum time)*
2. *Have you had any personal experiences with these barriers? If so, could you explain a little bit more about what happened?*
3. *How would you help your school overcome these barriers?*

**Q11 - How comfortable or confident are you using movement activities in the classroom?**

1. *Is there anything you feel you need to improve your capacity/ability to incorporate physical activity into the classroom?*
2. *Do you feel you would benefit from further training?*
3. *If so, how long should the training be and what should be covered?*
4. *How confident do you feel some of your other colleagues are to incorporate movement into a lesson?*

**Pupil Focus group topic guide**

- Ask for a teaching assistant in the room to help control behaviour and supervise.

**Research goals of the focus groups**

- What are children’s current perceptions of physical activity in primary school?
- What movement strategies or physical activity interventions have children experienced?
- What do children perceive as being negative or problematic when incorporating movement or physical activity into the classroom?
- What do children perceive as being positive or beneficial when incorporating movement or physical activity into the classroom?
- What movement strategies or physical activity interventions would work best from a child’s perspective?
- **Pitch our current design of the intervention to pupils and get feedback on this**

**Focus group**

1. ***Introduction (5mins)*** –

- Welcome, **introduction of researchers and children,** (make them feel as comfortable)
- Use of a fun **icebreaker activity** i.e. if you were an animal what animal would you be?, splat, age and favorite subject.
- Brief **introduction about the study** (use child friendly language), Make sure they are aware of the use of the recording device.
- Instructions regarding the focus group “**We are interested in hearing about what you think about using more body movement and getting up and moving more in your classrooms”** – stress that this does not just mean physical education lessons for example if they move to and from the whiteboard, around the room or outside etc. in math’s and science or any of their other lessons.

*Researcher script:*

“Right… now we all know each other we would really like to ask you a few questions about how you guys move about in the class and what you think about that! So from now on I would like to turn on this digital recorder so I can listen what we all say later and type it all up. Are you all ok for me to turn this on to record?

*Turn on recorder/ Address questions if anything raised.*

*Prompts*.

**Do you guys get to move around in the classroom?*

**when*

**where*

**how*

**what do you think about that?*

- We will ask if their school or teachers have tried any physical activity initiatives in the classroom, this may require prompts such as the Daily mile, Go Noodle etc. – what resources were used?

Q: Have your teachers done anything in particular that has got you up out of your seat? For example does your teacher get you to stand up, or to move more like walking around, jumping, or other movements during in class (but outside of PE)?

*Expansion Prompts:*

*What sort of things were these?

*How does your teacher get you to this?

*During which lessons?

*How often?

*When? – what points in the day or lesson do you move most?

*These little movement breaks, how long do they usually last for, do you know?

*What do you think about them?

Q: Aside from PE, what lessons are the most active at the moment?

*Expansion prompts:*

*what does your teacher get you to do in these lessons?

*How do they get you to do it?

*How long does it last for?

*What do you think about it?

* How much movement do you think is best?

Q: How do these bits of standing up and moving during class affect you??

*Expansions/ Clarification prompts:*

*Does is influence how you understand lesson content? or change your concentration? Mood?

*Can you tell me a bit more about how it influences/affects these things? Good/Bad?

*Is there anything else you’ve noticed that is different after moving if you think about how things were in class before moving?

Q: How about the others in class?

Expansion prompts:

*How long does it take everyone to settle back down into their seats and focus on the teacher again after having moved about?

*Is there anyone that takes longer than others? Anyone who acts differently from everyone else after these movement breaks?

*Any other positives or negatives experienced after the movement?

Q. Do you enjoy this? What do you like the most and least about this?

1. ***The following 5 mins –***

*So now we’ve talked about what you guys already do in class. We’d love to know what you think can help make the lessons even more active.*

*Q:*

*Expansion prompts.*

- How do you feel your teachers could include more movement into lessons or the school day?
- Do you have any specific ideas? – for a particular subject
- If you could design a lesson with lots of movement in it what would you do?
- Is there anything else you would like to say?
- If all avenues of possible discussion have been exhausted and we still have time left we may ask the children to draw pictures to help in their explanation. E.g. could you draw a quick picture about what your perfect 10 minutes of physical activity would look like? Or could you draw an image to show how you feel after 5 minutes of physical activity in the classroom.
- We will ask the children to explain the pictures they have drawn which will be recorded and transcribed. The researcher may also wish to use their own perceptions of the drawings to use in the analysis.

*Intervention ideas*

- What do you think about a 5 minute long physical activity in an hour lesson?
- Would you prefer a choice of what activity you do? – how could this be done?
- Easier for teachers to use software such as Go Noodle – do you like this?
- Do you like copying videos and dance moves in the classroom?
- At what points in the lesson do you get tired and need to move? – how many minutes in?
- How much time in a percentage do you think you spend on task in a lesson?
- Do you often get distracted in a lesson? How could this be stopped?
- Would you feel ok if researchers from the university observed your lesson or would this be too distracting to your lesson?

***Conclusion – about 5 minutes***

- Sum up what has been discussed, mention the positive aspects – check they are all happy, compliment and thank the children.
- How did you like talking about movement in classrooms?
- Is there anything important to you we haven’t mentioned?
- If you would like to follow any issues you have talked about, you can contact us (we will give the school our information and a poster with our contact details on)
